# West Nile in Italy: a new health challenge or human failure?

**DOI:** 10.1016/j.lanepe.2025.101440

**Published:** 2025-08-30

**Authors:** Francesco Branda, Giancarlo Ceccarelli, Marta Giovanetti, Fabio Scarpa, Massimo Ciccozzi

**Affiliations:** a Unit of Medical Statistics and Molecular Epidemiology, Università Campus Bio-Medico di Roma, Rome, Italy; b Genomics, AI, Bioinformatics, Infectious Diseases, Epidemiology Group (GABIE), Rome, Italy; c Department of Public Health and Infectious Diseases, University of Rome Sapienza, Rome, Italy; d Sciences and Technologies for Sustainable Development and One Health, Università Campus Bio-Medico di Roma, Italy; e Instituto Rene Rachou, Fundação Oswaldo Cruz, Minas Gerais, Brazil; f Department of Biomedical Sciences, University of Sassari, Sassari, Italy

West Nile virus (WNV), a mosquito-borne virus of the *Flaviviridae* family, has become one of the most significant arboviruses in Europe. Since the 1996 outbreak, WNV has expanded across the continent due to environmental, climatic, and anthropogenic factors such as urbanisation, land use changes, and rising summer temperatures.^1^, ^2^, ^3^ Italy is a key case study, with growing human cases and geographic spread—especially in the north—demonstrating the complex interaction of these factors.^4^ Despite expert warnings and health guidelines, preparedness remains inadequate. Vector control programmes, essential for preventing mosquito proliferation, are often delayed or neglected. Climate change, which should act as an early warning, is frequently overlooked, resulting in prolonged transmission seasons. Rather than proactive planning, the response to WNV has been reactive, lacking innovation such as biological control or environmentally sustainable strategies.

WNV now exemplifies the failure to turn past experiences into long-term action. The absence of a coordinated strategy based on continuous monitoring and ecological intervention raises concerns. Local management remains fragmented, often resorting to short-term measures like chemical pesticides, which harm ecosystems and biodiversity. Sustainable alternatives—such as natural mosquito predators or sterilisation techniques—are underutilised, despite their long-term benefits.

The surge in WNV cases in July 2025, surpassing previous seasons, calls for a systematic and science-based response rather than alarmism. As transmission occurs only through infected mosquitoes—not via direct human contact—control strategies must focus on reducing vector populations in a way that protects public health and the environment. This includes continuous monitoring of mosquitoes, birds, and animals to detect transmission hotspots and enable targeted interventions. Larval control should be prioritised over widespread adulticide use, as it is more sustainable and effective. Treating stagnant water, a key mosquito breeding ground, is critical. Innovative methods, such as introducing natural predators or deploying sterilised mosquitoes, should be expanded. Public education is equally vital—raising awareness about repellents, mosquito nets, and exposure reduction during peak seasons.

Ultimately, WNV management is not just a health issue—it reflects systemic challenges in adapting to climate and environmental change. Until policies shift toward long-term, integrated strategies involving science, sustainability, and public engagement, one question remains: how much longer must we wait before WNV management is treated as a strategic public health priority?

## Contributors

**Francesco Branda**: Conceptualization, Investigation, Writing–Original Draft, Writing–Review & Editing. **Giancarlo Ceccarelli**: Investigation, Writing–Original Draft, Writing–Review & Editing. **Marta Giovanetti**: Investigation, Writing–Original Draft, Writing–Review & Editing. **Fabio Scarpa**: Investigation, Writing–Original Draft, Writing–Review & Editing. **Massimo Ciccozzi:** Conceptualization, Supervision, Validation, Writing–Original Draft, Writing–Review & Editing.

## Declaration of interests

The authors declare that they have no known competing financial interests or personal relationships that could have appeared to influence the work reported in this paper.
